# Older Vietnamese Have the Highest Prevalence of Disability Compared to White and Other Asian Groups

**DOI:** 10.1093/geroni/igab046.2091

**Published:** 2021-12-17

**Authors:** Hoang Nguyen, Christina Miyawaki, Kyriakos Markides

**Affiliations:** 1 University of Texas Medical Branch at Galveston, Galveston, Texas, United States; 2 University of Houston, Houston, Texas, United States; 3 University of Texas Medical Branch, Galveston, Texas, United States

## Abstract

The COVID-19 pandemic has highlighted the vulnerability of older adults with pre-existing health conditions and disabilities. A 2011 study reported that Asian older adults had lower prevalence of disability compared to non-Hispanic white. We revisited the estimate a decade later using the recently released 2015-2019 Public Use Microdata Sample (PUMS) from the American Community Survey (ACS). We estimated the prevalence of six types of disability in adults aged 60 years and older who self-identified as Vietnamese, Chinese, Filipino, Japanese, Korean, Asian Indian, or non-Hispanic White. We also compared the risk for each disability type between Vietnamese and non-Hispanic White (reference group) using the adjusted (age, sex, marital status, education and poverty level) odds ratios. All analyses used survey weights for point estimate and the jackknife method for standard error. Significantly higher prevalence of limitations in independent living, self-care, cognitive function, and blindness were reported by Vietnamese than by non-Hispanic White. Vietnamese also had the highest prevalence in all six types of disability of the Asian groups examined. The adjusted odds ratio of limitations in independent living, self-care, and cognitive function was significantly higher for Vietnamese than non-Hispanic White. These findings suggest a possible negative outcome trend with the aging of the Vietnamese population. We discuss the historical accounts of Vietnamese in the United States as war refugees and family reunion migrants, provide possible explanations for these new findings including changing demographic structures, and make recommendations for policy and practice that incorporate existing social and cultural resources in the Vietnamese community.

